# P049: High rate of MRSA respiratory tract colonisation in HIV-positive children in Cambodia during 2004-2012

**DOI:** 10.1186/2047-2994-2-S1-P49

**Published:** 2013-06-20

**Authors:** J Sokolova, N Kulkova, A Liskova, A Streharova, A Shahum, G Benca, V Krcmery

**Affiliations:** 1 Department of Laboratory Medicine, Trnava University in Trnava, Trnava, Slovakia; 2 Laboratory of Molecular Microbiology, St. Elisabeth University, Bratislava, Slovakia; 3 Department of Public Health, Trnava University in Trnava, Trnava, Slovakia; 4 St. Maximilian Kolbe Clinic, Phnom Penh, Cambodia

## Introduction

Children attending child care centres are at increased risk of infections, including those caused by MRSA (methicillin-resistant *S. aureus*).

## Objectives

The aim of this study was to evaluate MRSA colonization among HIV-infected children in two orphanages in Cambodia during period 2004-2012 and to assess risk of spreading MRSA within these specific health-care facilities.

## Methods

Totally 137 HIV positive children (39,4% male; median age 7, IQR= 5-9) were enrolled in our HIV programmes during 2004-2012 (follow up 51±28 months). Every 6 months, respiratory swabs were obtained, followed by organisms’ identification and susceptibility testing according to CLSI guidelines.

## Results

We have collected 586 respiratory swabs positive for bacteria. Considering overall aetiology, *S. aureus* was predominant (178 isolates; 30,4%), followed by *Str. pneumoniae* (103; 17,6%) and *K. pneumoniae* (99; 16,9%). *M. catarrhalis* and *H. influenzae* were present in less extent (35; 6,0% and 20; 3,4%, respectively). In relation to resistance, we found out oxacillin resistance (phenotype MRSA) to be the most prevalent among *S. aureus* (112 isolates; 63%) and resistance to other classes of antibiotics was high in this group of pathogens, too (e.g. clindamycin-59%; erythromycin-4,3%). However, susceptibility to some other antibiotics, such as vancomycin, linezolid, ciprofloxacin and co-trimoxazole was very well (100%; 100%; 96% and 95%, respectively). Typing of hypervariable region of methicillin resistance gene (HVR-*mecA* typing) among selected MRSA isolates revealed six different HVR-types, with the type I being the most frequent (41,2%).

## Conclusion

Despite the TMP/STX prophylaxis, we found out high rates of MRSA colonisation but resistance to this antibiotic remained rare. This is supporting assumption that prophylaxis is decreasing exposure to other pathogens and consequently, selective pressure of antibiotics, too. Diversity among HVR-*mecA*-types indicates that MRSA in our study were not spread clonally in this specific healthcare facility.

## Disclosure of interest

None declared

